# Supplementary material for: Volatilomics Analysis of Jasmine Tea during Multiple Rounds of Scenting Processes
Source: Foods. 2023 Feb 14;12(4):812. doi: 10.3390/foods12040812 (PMC9956320; doi:10.3390/foods12040812)
Supplement: Supplementary file 1 [file foods-12-00812-s001.zip › Supplementary Figures.pdf]

foods

Supplementary Figures

Volatilomics Analysis of Jasmine Tea During Multiple Rounds of Scenting Processes

Cheng Zhang[1,2,†], Chengzhe Zhou[1,2,3,†], Caiyun Tian[1,2], Kai Xu[1,2], Zhongxiong Lai[1,3], Yuling Lin[1,3], and Yuqiong Guo[1,2,*]

1 College of Horticulture, Fujian Agriculture and Forestry University, Fuzhou 350002, China
2 Tea Industry Research Institute, Fujian Agriculture and Forestry University, Fuzhou 350002, China
3 Institute of Horticultural Biotechnology, Fujian Agriculture and Forestry University, Fuzhou 350002, China
* Correspondence: guoyq828@163.com
† These authors contributed equally to this work.

This PDF file includes: Supplementary Figures S1–S8

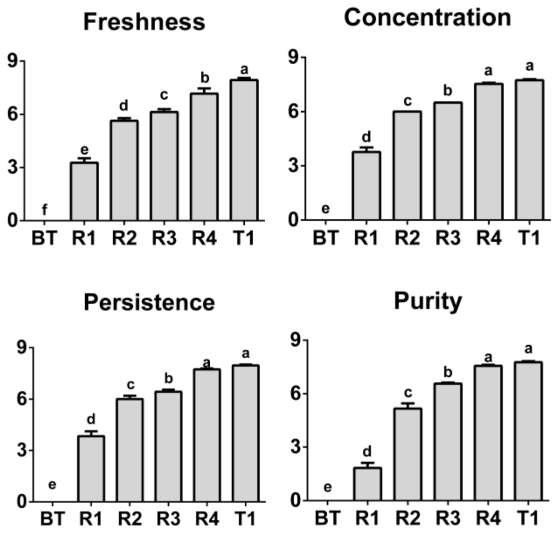

Figure S1. The significant difference analysis of taste attributes in base tea and jasmine tea with different rounds of scenting process. BT: base tea; R1: jasmine tea with one round of scenting process; R2: jasmine tea with two rounds of scenting processes; R3: jasmine tea with three rounds of scenting processes; R4: jasmine tea with four rounds of scenting processes; T1: jasmine tea with four rounds of scenting processes and Tihua process. The various small letters represent significant differences ($p < 0.05$).

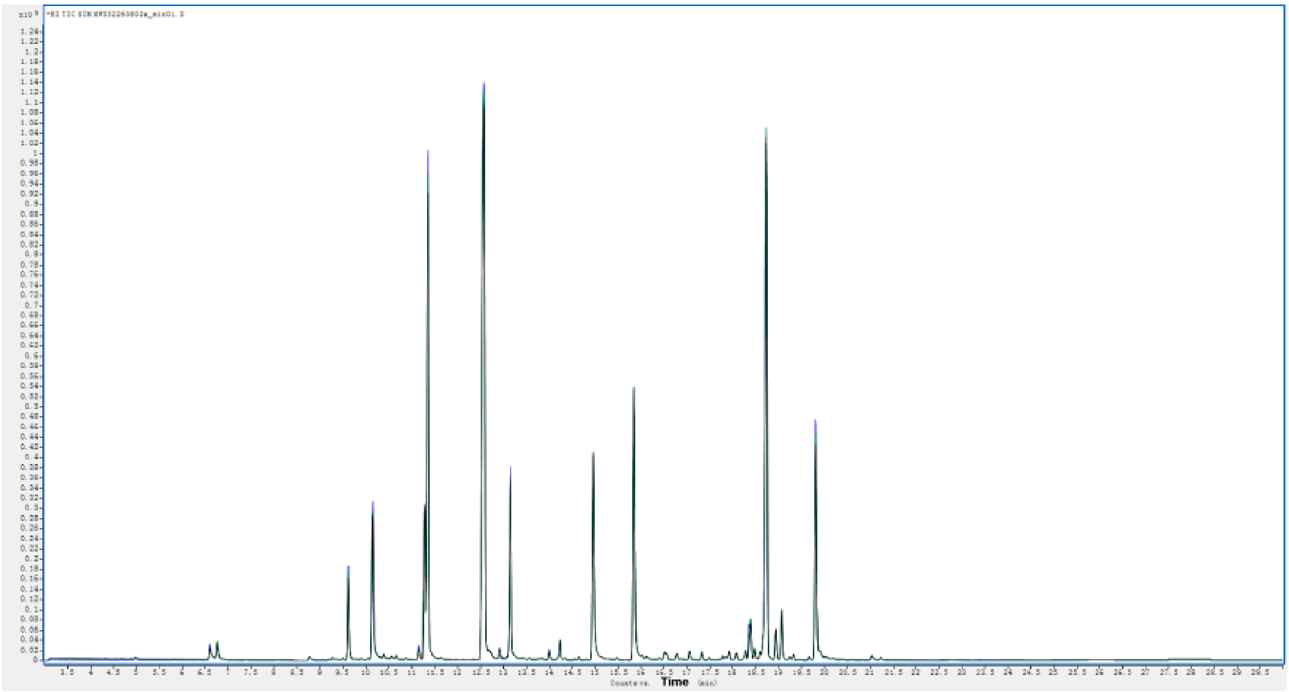

Figure S2. Total ion chromatogram of mass spectrometry analysis of the mixed sample for quality control.

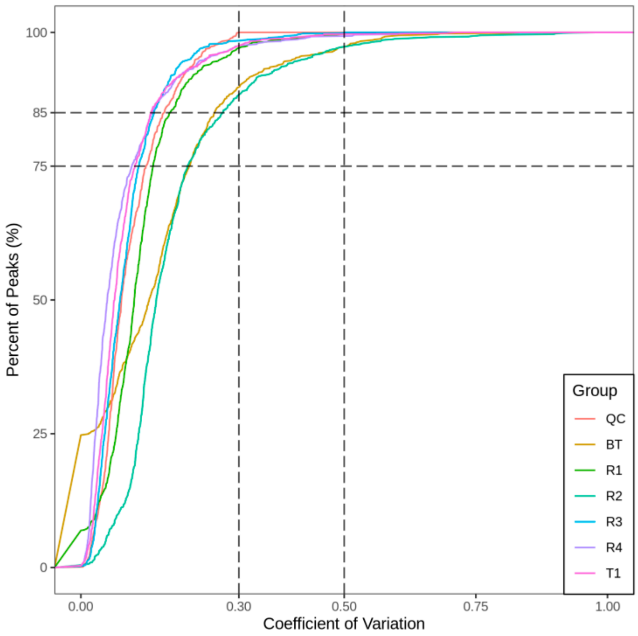

Figure S3. The Coefficient of Variation for all the groups. QC: quality control; BT: base tea; R1: jasmine tea with one round of scenting process; R2: jasmine tea with two rounds of scenting processes; R3: jasmine tea with three rounds of scenting processes; R4: jasmine tea with four rounds of scenting processes; T1: jasmine tea with four rounds of scenting processes and Tihua process.

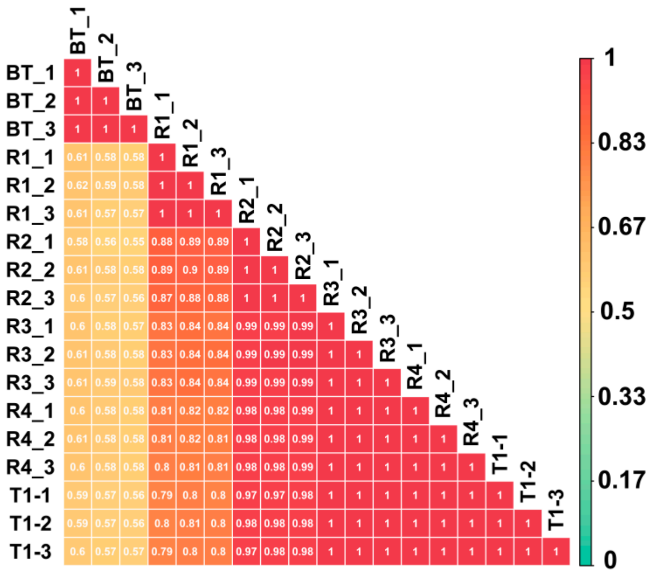

Figure S4. The correlation analysis using Pearson's Correlation Coefficient (PCC) between samples and groups. The numbers in rectangles represent PCCs. BT: base tea; R1: jasmine tea with one round of scenting process; R2: jasmine tea with two rounds of scenting processes; R3: jasmine tea with three rounds of scenting processes; R4: jasmine tea with four rounds of scenting processes; T1: jasmine tea with four rounds of scenting processes and Tihua process.

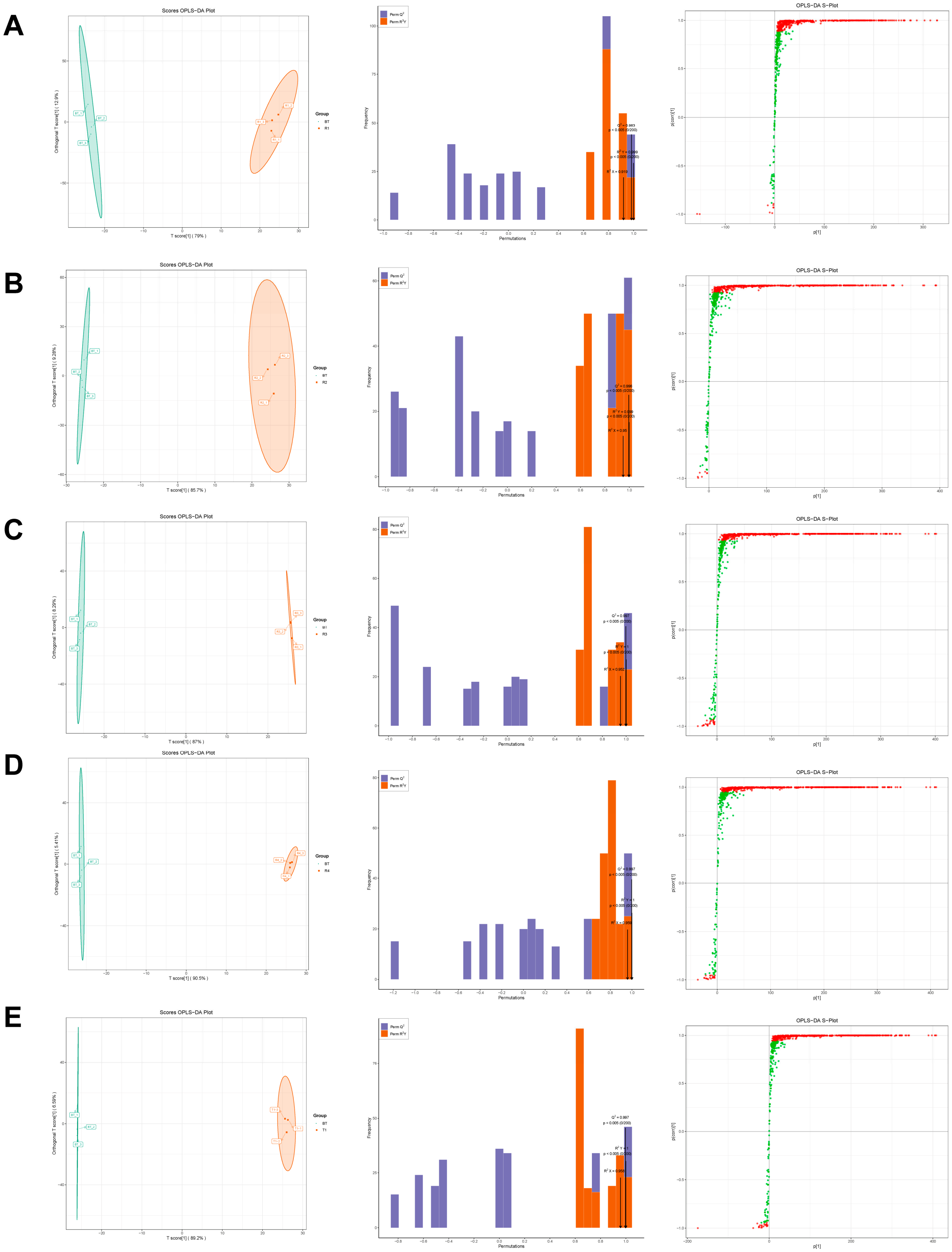

Figure S5. OPLS-DA score plots (left), permutation tests (middle), and S-plots of each pairwise comparison between the base tea and jasmine tea samples. The compared combinations were BT *vs.* R1 (A); BT *vs.* R2 (B); BT *vs.* R3 (C); BT *vs.* R4 (D); and BT *vs.* T1

(E). BT: base tea; R1: jasmine tea with one round of scenting process; R2: jasmine tea with two rounds of scenting processes; R3: jasmine tea with three rounds of scenting processes; R4: jasmine tea with four rounds of scenting processes; T1: jasmine tea with four rounds of scenting processes and Tihua process.

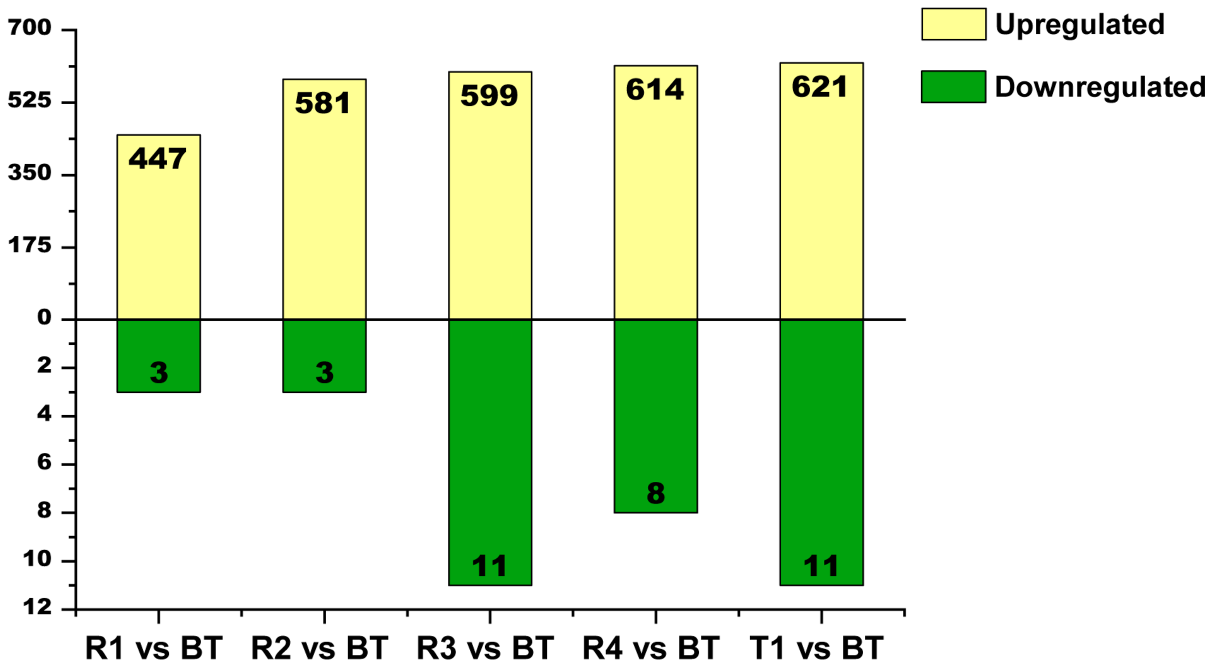

Figure S6. Number of differential metabolites of each pairwise comparison of base tea and jasmine tea samples. BT: base tea; R1: jasmine tea with one round of scenting process; R2: jasmine tea with two rounds of scenting processes; R3: jasmine tea with three rounds of scenting processes; R4: jasmine tea with four rounds of scenting processes; T1: jasmine tea with four rounds of scenting processes and Tihua process.

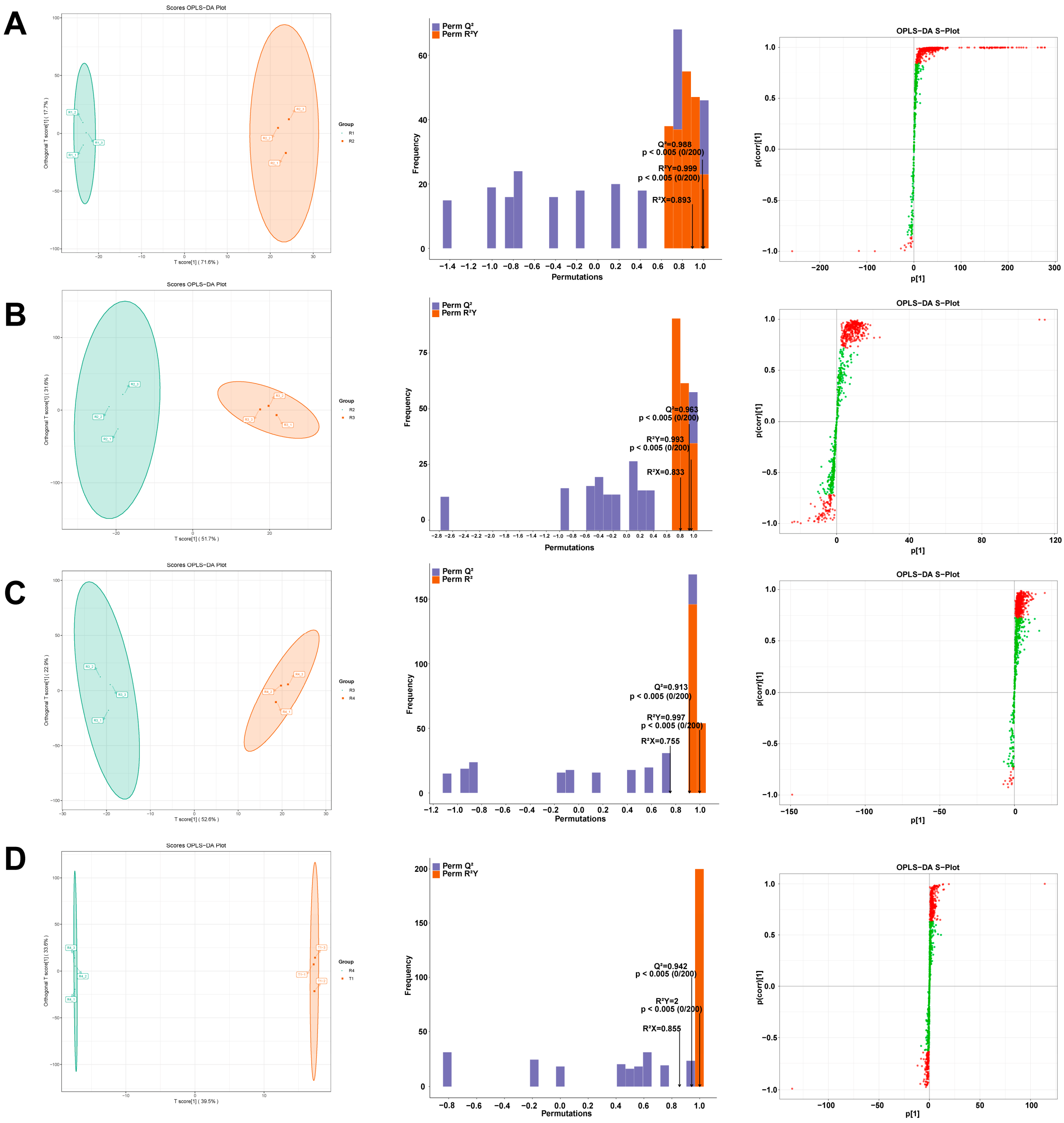

Figure S7. OPLS-DA score plots (left), permutation tests (middle), and S-plots of each pairwise comparison between the jasmine tea samples with different rounds of scenting processes. The compared combinations were R1 *vs.* R2 (A); R2 *vs.* R3 (B); R3 *vs.* R4 (C); and R4 *vs.* T1 (D). BT: base tea; R1: jasmine tea with one round of scenting process; R2: jasmine tea with two rounds of scenting processes; R3: jasmine tea with three rounds of scenting processes; R4: jasmine tea with four rounds of scenting processes; T1: jasmine tea with four rounds of scenting processes and Tihua process

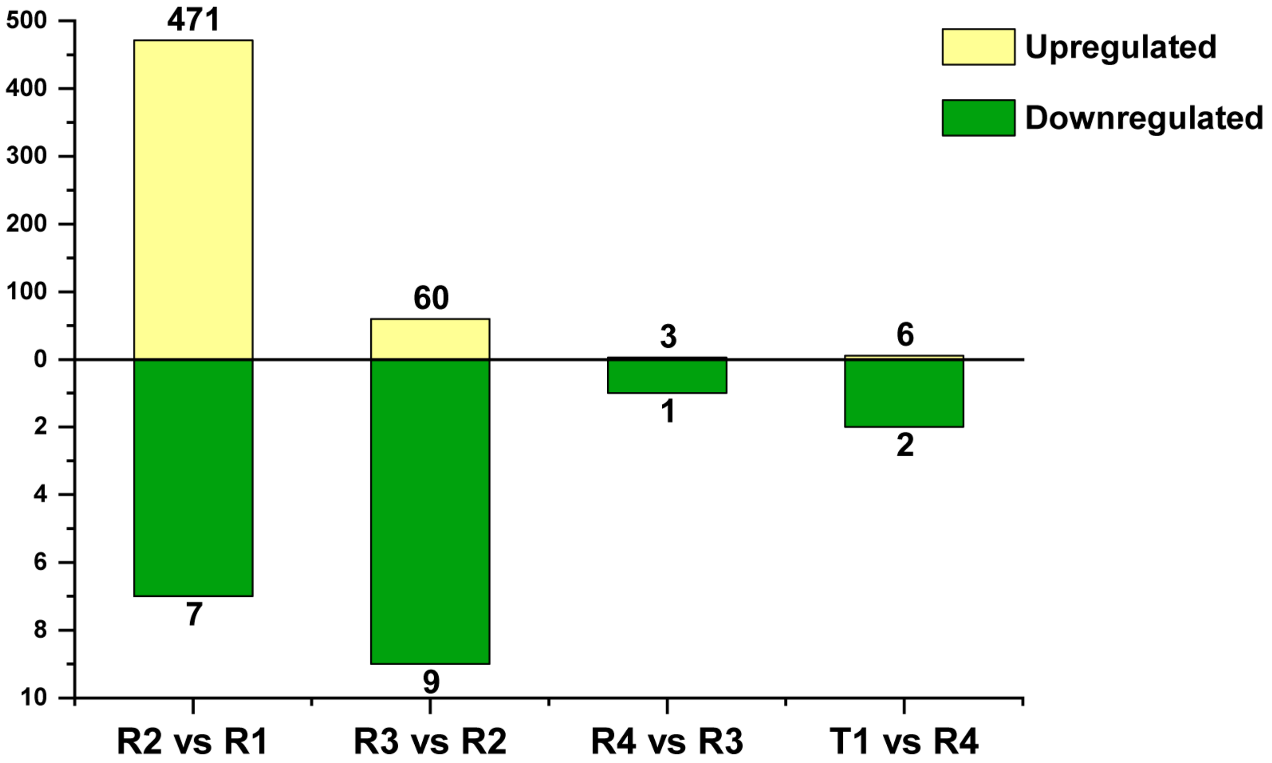

Figure S8. Number of differential metabolites of each pairwise comparison of jasmine tea during consecutive scenting. R1: jasmine tea with one round of scenting process; R2: jasmine tea with two rounds of scenting processes; R3: jasmine tea with three rounds of scenting processes; R4: jasmine tea with four rounds of scenting processes; T1: jasmine tea with four rounds of scenting processes and Tihua process.
